# A Case Report on the Efficacy of Trastuzumab Emtansine in a Patient With Human Epidermal Growth Factor Receptor 2 Exon 20-Mutated Adenocarcinoma of the Lung

**DOI:** 10.7759/cureus.38271

**Published:** 2023-04-28

**Authors:** Arun Warrier, Anu George, Vipulkumar Thummar, Priya Mehta

**Affiliations:** 1 Medical Oncology, Aster Medcity, Kochi, IND; 2 Medical Affairs, Zydus Lifesciences Limited, Ahmedabad, IND

**Keywords:** mutation, exon 20, lung cancer, her2, t-dm1

## Abstract

Lung cancer is the foremost reason for cancer-related mortality among men and women. The ultimate goal of patient supervision post-diagnosis for advanced cases is to improve survival and quality of life with minimal treatment-associated side effects. With advancements in genomic medicine and a better understanding of cell signaling pathways, many actionable gene mutations have been identified in lung carcinoma, which drastically improve survival outcomes. Mutations in *human epidermal growth factor receptor 2* (*HER2*) and *epidermal growth factor receptor* together are observed in nearly 1-3% of cases and act as an oncogenic driver. In the case of *HER2*-mutant lung cancers, there are limited approved agents, and the treatment represents a critical unmet medical need because of the poorer survival outcomes compared to patients with additional oncogenic drivers. The recent standard of care of treatment is chemotherapy, but reports suggest that compared with cytotoxic chemotherapy, patients receiving HER2-directed therapies have relatively longer median survival duration. Here, we report a case of *HER2 exon 20*-mutated metastatic lung adenocarcinoma patient who received trastuzumab emtansine in the third-line setting and achieved durable disease control.

## Introduction

Lung cancer is the foremost reason for cancer-related mortality among men and women. The ultimate goal of patient supervision after the diagnosis for advanced cases is to improve survival and quality of life with minimal treatment-associated side effects. Enhanced research on the molecular pathways driving cancer in non-small-cell lung cancer (NSCLC) steered advances in targeted therapy with molecular pathway-specific agents in cancer cells [1-3].

With advancements in genomic medicine and a better understanding of cell signaling pathways, many actionable gene mutations have been identified in lung carcinoma. Drugs targeting pathways such as epidermal growth factor receptor (EGFR), anaplastic lymphoma kinase (ALK), and programmed death-ligand 1 (PD-L1) have improved survival outcomes in lung adenocarcinoma [2]. Other targetable mutations are *MET*, *AKT*, *BRAF*, *PIK3CA*, *RET*, *KRAS*, and *ROS *rearrangements [2]. In NSCLC, mutations in *human epidermal growth factor receptor 2* (*HER2*) and *EGFR* together are observed in nearly 1-3% of cases and act as an oncogenic driver involving small in-frame insertions and point mutations in exon 20 [1-3].

Previous reports have suggested a poorer prognosis in *HER2*-mutant advanced lung cancer patients compared to other oncogenic drivers [2-4]. In the case of *HER2*-mutant lung cancers, there are limited approved agents yet, though reports suggest treatment responses toward both chemotherapies and HER2- targeted therapies. As per the real-world study of a French cohort, a median overall survival of 10.7 months was observed in stage IV NSCLC patients harboring *HER2* mutation treated with standard treatment per guidelines [5].

According to Patil et al. (2019), patients with *EGFR* and *HER2* mutations have variable efficacy in EGFR and HER2-directed therapy. According to the data, 37% (7/19) of patients received HER2-directed therapy (which includes tyrosine kinase inhibitors (TKIs), HER2 monoclonal antibodies, and HER2 antibody-drug conjugates) during their treatment. Results suggest that in comparison with the patients receiving cytotoxic chemotherapy, patients receiving HER2-directed therapies had relatively longer median survival duration (65 versus 29 months) [6].

A case series of *HER2*-mutant lung cancers suggested higher response rates of up to 50% in patients who used trastuzumab in combination with chemotherapy [2,7]. Based on such reports and studies, the role of trastuzumab is impossible to determine as it was always combined with chemotherapy backbone. Despite the availability of multiple HER2-directed agents, there is very limited globally approved targeted therapy for *HER2*-mutant lung cancers, for example, trastuzumab deruxtecan.

Ado-trastuzumab emtansine (T-DM1) is an HER2-targeted antibody-drug conjugate. It has trastuzumab linked with the antimicrotubular agent emtansine. It is an approved agent for HER2-amplified or overexpressing metastatic breast cancer patients [8]. The response toward T-DM1 has also been reported in *HER2*-mutant lung cancer patients [9].

To date, very little reporting has been done as case reports and clinical trials, and the results suggest a role of HER2-directed therapy with promising tumor response. Previous reports also stated that *HER2*-mutated patients are resistant to EGFR TKIs, but part of them is sensitive to both HER2 inhibitors and dual EGFR/HER2 inhibitors [10].

Here, we report a case of *HER2 exon 20*-mutated metastatic adenocarcinoma lung who received T-DM1 in the third-line setting and achieved durable disease control.

## Case presentation

A 50-year-old male non-smoker patient with no comorbidities was evaluated for complaints of headache, gait imbalance, history of a fall, and blurring of vision in the past month at a tertiary care center. MRI of the brain suggested a vermian contrast-enhancing lesion with mass effect.

The patient underwent a midline suboccipital craniotomy and excision in August 2019. Postoperative histopathology examination (HPE) suggested a cerebellar vermian lesion with metastasis from pulmonary papillary adenocarcinoma. Positron emission tomography-computed tomography (PET-CT) scan revealed a lesion in the left upper and lower lobe of the lung with non-fluorodeoxyglucose (FDG)-avid mediastinal nodes, and the patient was diagnosed with carcinoma of the left lung, CT2N0M1, stage IV. Further analysis was done with next-generation sequencing (NGS), and the test was found to be negative for EGFR, ALK, ROS, and PD-L1 (<1%), while it was positive for ERBB2 and exon 20 mutation (Tyr772_Ala775dup).

The patient was planned for stereotactic body radiation therapy for brain and lung lesions and completed radiation with 30 Gy in five fractions in September 2019. He received three weekly chemotherapy treatments with pemetrexed-carboplatin until November 2019, and a reassessment was done with a CT of the thorax after four cycles in December 2019. Results showed good treatment response in terms of stable disease, and an MRI of the brain showed no residual/recurrent disease. Hence, he was kept on maintenance pemetrexed and underwent 10 cycles until June 2020.

PET-CT in July 2020 suggested disease progression with FDG uptake in the left lung, mediastinal nodes, and new lesions in the brain. He received an excision followed by radiotherapy for the brain and was then put on docetaxel and completed eight cycles of chemotherapy until December 2020.

PET-CT in January 2021 suggested persistent cerebellar lesions but stable disease. The patient was kept under follow-up with CT of the thorax and MRI of the brain at regular intervals, which showed stable disease for six months.

**Figure 1:**
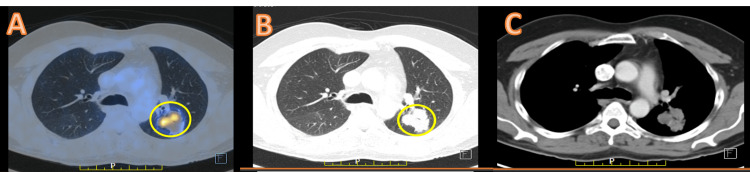
(A-C) Positron emission tomography-computed tomography scan suggestive of local disease progression with an increase in the size and metabolic activity of the left lung in the upper lobe.

**Figure 2:**
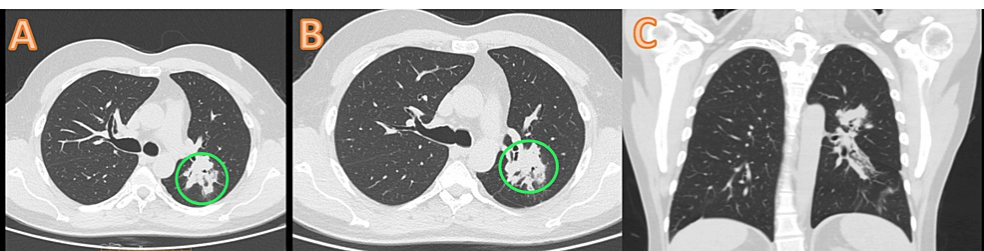
(A-C) Mild interval decrease in the solid component (non-measurable) of the left lung mass with a partial replacement by ground-glass densities and fibrosis after ado-trastuzumab emtansine treatment.

Results of PET-CT in September 2021 suggested disease progression, as well as an increase in lung lesions with a stable cerebellar lesion, as shown in Figure 1. Thus, the options of T-DM1 and pemetrexed-carboplatin were discussed with the patient and his family. The patient decided to proceed with T-DM1 treatment after a thorough understanding of the medical condition and an in-depth discussion with the clinical team. As the patient had access to an approved T-DM1 biosimilar option available in the country, the same was selected, and he was started on T-DM1 in view of ERBB2/HER2 positivity. After four cycles of T-DM1 (dose 200 mg q3w), contrast-enhanced CT of the thorax was done and showed stable disease, and he further received four additional cycles of T-DM1 until February 2022 (Figure 2).

PET-CT assessment was again done in March 2022, which suggested disease progression with an increase in the size of the lung lesion. Therefore, he was given radiation therapy using the three-dimensional conformal radiation therapy technique at a dose of 45 Gy in 15 fractions to the lung lesion until April 2022. He has been advised to continue chemotherapy with oral etoposide and remains under supportive care.

## Discussion

Targeted therapy for NSCLC toward specific molecular alterations such as ALK rearrangements or *EGFR* mutations represents an incredible advancement in therapy [11,12]. Recent research is focussed on identifying and developing targeted therapy for other molecular subtypes. As NGS has become more accessible and regularly used in the preliminary assessment of lung cancers, earlier identification of *HER2 *mutations is possible in the clinical course.

In NSCLC, HER2 gets activated via three mechanisms, namely, gene mutation (1-4% of cases), gene amplification (2-5%), and protein overexpression (2-30%) [13]. These three subgroups have different clinical features and prognoses and should be treated differently. Exon 20 insertions are the most common *HER2* mutation and have previously been shown to be mutually exclusive to other mutations such as *EGFR*, *KRAS*, *NRAS*, *ALK*, *PI3KCA*, and *BRAF* [1].

There is a critical unmet medical need in the context of *HER2*-mutant lung cancer treatment because of the poorer survival outcomes compared to the patients with additional oncogenic driver mutations [3,4]. The existing standard-of-care treatment is platinum-based chemotherapy along with maintenance pemetrexed which provides the maximum advantage [14].

Fewer studies have been done to assess the efficacy of anti-HER2 agents in *HER2*-mutant or positive NSCLC patients. However, unlike in breast and gastric cancers, anti-HER2 agents, such as trastuzumab, pertuzumab, or non-selective TKIs, have shown little effect in lung adenocarcinoma [15]. Initially, in lung cancer patients, HER2-targeted therapy was used based on HER2 protein expression identified by immunohistochemical testing [16], where inhibition of HER2 signaling leads to antitumor activity and antibody-dependent cell-mediated cytotoxicity, as in the case of breast cancers.

However, in case of low or undetectable levels of HER2 protein expression, mass spectrometric analysis is used to confirm *HER2 *mutants and responders toward T-DM1. Overexpression of HER3 protein has been seen in a few patients, signifying the potential role of HER3 dimerization among *HER2 *mutants. Superior binding and internalization of trastuzumab might occur because of this mechanism via better phosphorylation and receptor ubiquitination [17]. Thus, T-DM1 attached to mutant *HER2 *might have a higher grade of cellular internalization compared to wild-type receptors, irrespective of the amount of HER2 protein, making it a promising therapeutic agent.

Per the study by Li et al.( 2018), a phase II clinical trial including 18 patients of NSCLC with *HER2 *mutations and heavily pretreated with metastatic disease state, a 44% (95% CI = 22-69%) objective response rate (ORR) was observed with T‑DM1, and nearly five months (95% CI = 3-9 months) of median progression-free survival (PFS) was observed. Additionally, T‑DM1 was accepted well among patients, with the common adverse events observed including infusion reactions, anemia, thrombocytopenia, and transaminitis of severity grade 1 or 2 [18].

These results suggest significant therapeutic progress in the context of a decade-long history of negative clinical trials targeting HER2 in lung tumors. Most of them were studies of a combination of chemotherapy with HER2 agents, which did not show the advantage of HER2 agents over chemotherapy. Based on the study results, the National Comprehensive Cancer Network (NCCN) 2018 guidelines recommended the treatment of *HER2*‑mutant NSCLCs with T‑DM1 (category 2A), and the same remains in the latest NCCN guidelines as well [19]. A supplementary extension study of T-DM1 in both HER2- amplified and HER2-mutant solid tumors is ongoing (NCT02675829).

In the EUHER2 cohort study, a single patient was found to have a rapid response toward T-DM1, as reported in a separate paper [9]. The collated results of trastuzumab-based treatment and T-DM1 suggest a relative risk of 50.9% and PFS of 4.8 months (95% CI = 3.4; 6.5).

According to Huang et al. (2020), an ORR of 44% (95% CI = 25-63%) was observed in the *HER2 *gene mutated and amplified and/or overexpressed subtype, which was identical to the *HER2* gene mutated alone subset (44% vs. 41%). While patients with HER2 overexpression along with mutations revealed lower ORR compared to *HER2 *mutations alone [27% (95% CI = 0-54%) vs. 41% (95% CI = 11-70%)]. This indicates that HER2 overexpression might have a negative influence on *HER2 *mutations in terms of response toward T-DM1 [20].

Markedly, patients having all three HER2 abnormalities presented with a higher ORR compared to HER2 mutations alone or overexpression collated with mutations [80% (95% CI = 50-100%) vs. 41% (95% CI = 11-70%) vs. 27% (95% CI = 0-54%), respectively]. *HER2 exon 20* insertion was the most commonly reported mutation, associated with an ORR of 40% (95% CI = 18-61%). Nevertheless, no meaningful conclusions can be drawn about the type of mutation connected with T-DM1 effectiveness due to the small number of study subjects [20].

The discrepancy among the data can be partly justified by the smaller sample size of the majority of studies as well as the study designs, i.e., single arm and single center. Overall, it remains a gray area regarding the concomitance of different HER2 alterations and their overall effects on the efficacy of T-DM1 [20].

## Conclusions

In lung cancer, *HER2 *is found to be an effective targetable driver mutation. We recommend testing HER2 along with the testing of other rare, potentially targetable genetic alterations for patients whose tumors are observed to be negative for *EGFR*, *ALK*, and *ROS1*. Presently available advanced HER2-targeted agents might provide an added opportunity for the treatment of such patients.

T-DM1 is now approved as a treatment of choice for *HER2*-mutated NSCLC patients according to the latest NCCN guidelines. This report provides evidence of a durable response of approximately six months with this drug in the third-line setting. This report justifies the need for further studies to evaluate response to T-DM1, especially in *HER2*-mutated lung carcinoma patients progressing on routine chemotherapy.

## References

[1] Arcila ME, Chaft JE, Nafa K (2012). Prevalence, clinicopathologic associations, and molecular spectrum of ERBB2 (HER2) tyrosine kinase mutations in lung adenocarcinomas. Clin Cancer Res.

[2] Mazières J, Peters S, Lepage B (2013). Lung cancer that harbors an HER2 mutation: epidemiologic characteristics and therapeutic perspectives. J Clin Oncol.

[3] Pillai RN, Behera M, Berry LD (2017). HER2 mutations in lung adenocarcinomas: a report from the Lung Cancer Mutation Consortium. Cancer.

[4] Kris MG, Johnson BE, Berry LD (2014). Using multiplexed assays of oncogenic drivers in lung cancers to select targeted drugs. JAMA.

[5] Auliac JB, Dô P, Bayle S (2019). Non-small cell lung cancer patients harboring HER2 mutations: clinical characteristics and management in a real-life setting. Cohort HER2 EXPLORE GFPC 02-14. Adv Ther.

[6] Patil T, Mushtaq R, Marsh S (2020). Clinicopathologic characteristics, treatment outcomes, and acquired resistance patterns of atypical EGFR mutations and HER2 alterations in stage IV non-small-cell lung cancer. Clin Lung Cancer.

[7] Mazières J, Barlesi F, Filleron T (2016). Lung cancer patients with HER2 mutations treated with chemotherapy and HER2-targeted drugs: results from the European EUHER2 cohort. Ann Oncol.

[8] Verma S, Miles D, Gianni L (2012). Trastuzumab emtansine for HER2-positive advanced breast cancer. N Engl J Med.

[9] Weiler D, Diebold J, Strobel K, Aebi S, Gautschi O (2015). Rapid response to trastuzumab emtansine in a patient with HER2-driven lung cancer. J Thorac Oncol.

[10] Zhang X, Lv J, Wu Y (2020). HER2 exon 20 insertion mutations in lung adenocarcinoma: case series and response to pyrotinib. Front Oncol.

[11] (2023). National Comprehensive Cancer Network. NCCN clinical practice guidelines in oncology. Non-small cell lung cancer. https://www.nccn.org/guidelines/guidelines-process/about-nccn-clinical-practice-guidelines.

[12] Novello S, Barlesi F, Califano R (2016). Metastatic non-small-cell lung cancer: ESMO Clinical Practice Guidelines for diagnosis, treatment and follow-up. Ann Oncol.

[13] (2014). Comprehensive molecular profiling of lung adenocarcinoma. Nature.

[14] Eng J, Hsu M, Chaft JE, Kris MG, Arcila ME, Li BT (2016). Outcomes of chemotherapies and HER2 directed therapies in advanced HER2-mutant lung cancers. Lung Cancer.

[15] Krug LM, Miller VA, Patel J (2005). Randomized phase II study of weekly docetaxel plus trastuzumab versus weekly paclitaxel plus trastuzumab in patients with previously untreated advanced nonsmall cell lung carcinoma. Cancer.

[16] Ricciardi GR, Russo A, Franchina T (2014). NSCLC and HER2: between lights and shadows. J Thorac Oncol.

[17] Scaltriti M, Verma C, Guzman M (2009). Lapatinib, a HER2 tyrosine kinase inhibitor, induces stabilization and accumulation of HER2 and potentiates trastuzumab-dependent cell cytotoxicity. Oncogene.

[18] Li BT, Shen R, Buonocore D (2018). Ado-trastuzumab emtansine for patients with HER2-mutant lung cancers: results from a phase II basket trial. J Clin Oncol.

[19] Ettinger DS, Aisner DL, Wood DE (2018). NCCN guidelines insights: non-small cell lung cancer, version 5.2018. J Natl Compr Canc Netw.

[20] Huang X, Jin R, Lou L (2020). The efficacy of ado-trastuzumab emtansine in patients with ERBB2-aberrant non-small cell lung cancer: a systematic review. Transl Cancer Res.

